# On the Contribution of Binocular Disparity to the Long-Term Memory for Natural Scenes

**DOI:** 10.1371/journal.pone.0049947

**Published:** 2012-11-15

**Authors:** Matteo Valsecchi, Karl R. Gegenfurtner

**Affiliations:** Abteilung Allgemeine Psychologie, Justus-Liebig-Universität, Giessen, Germany; National Institute of Mental Health, United States of America

## Abstract

Binocular disparity is a fundamental dimension defining the input we receive from the visual world, along with luminance and chromaticity. In a memory task involving images of natural scenes we investigate whether binocular disparity enhances long-term visual memory. We found that forest images studied in the presence of disparity for relatively long times (7s) were remembered better as compared to 2D presentation. This enhancement was not evident for other categories of pictures, such as images containing cars and houses, which are mostly identified by the presence of distinctive artifacts rather than by their spatial layout. Evidence from a further experiment indicates that observers do not retain a trace of stereo presentation in long-term memory.

## Introduction

Human observers possess an astonishing long-term memory for images of objects and scenes. Early studies showed that observers can quite accurately recognize the gist of as many as 10.000 pictures of objects and scenes [1], [2].

The long-term memory for scenes, especially if enough processing time is available, is largely mediated by their semantic content. Observers can quickly build a conceptual representation of the scene [3] and, if enough time is available for encoding (e.g., [4]), this representation is consolidated in short-term memory and eventually transferred to long-term memory. A strong evidence for the conceptual encoding of scenes comes from the fact that observers are more likely to produce false recognitions when they encounter a scene conceptually related to the memorized one [5]. Nonetheless, visual memory for scenes has been shown to be resistant to interference ([6], but see [7]), and above all, there is evidence that observers can recognize specific instances of objects within a category even after learning thousands of items, visual long-term memory is thus potentially quite detailed [8]. The visual and conceptual codes for natural images coexist in long-term memory and have similar decay times, as demonstrated by the interference effects of visually and conceptually related distractors [9]. As far as scenes are concerned, the question arises as to what specific visual features are stored in memory and to what extent they contribute to the successful recognition of the scene.

The role of chromatic information has been assessed in a number of studies. In particular, Wichmann, Sharpe and Gegenfurtner [10] showed that images of scenes presented in color are remembered better than grayscale images. By manipulating the presence of color selectively in the encoding and in the recognition phase and by manipulating the exposure time of the images in the encoding phase they were able to show that color is stored in memory, besides contributing to the early perceptual processing of the image in the encoding phase. The role of color in the long-term memory for scenes was confirmed by Spence, Wong, Rusan and Rastegar [11] and Yao and Einhäuser [12]. Partially conflicting evidence has instead been reported by Nijboer, Kanai, de Haan and van der Smagt [13]. In their study they found evidence that the presence of color might actually hamper the fast encoding of natural scenes, specifically if the image has a meaningful gist.

Another visual dimension that has been shown to contribute to the memory for scenes is temporal change, and specifically motion. Dynamic scenes are remembered better [14], although this dynamic superiority effect seems to be mainly related to the preferential processing of specific dynamic objects within the scene rather than to a direct memorization of object motion [15]. Relatedly, multiple studies have investigated the role of observer motion in the memory for scene layout. Observer motion due to active navigation in an indoor scene was found to slightly improve performance in a recognition task as compared to passive viewing of static images [16]. Other studies however indicate that snapshot viewing might be sufficient to support recognition of scenes through which the observer moves [17].

Beyond luminance, chromaticity and motion, our visual system has access to another low-level feature when faced with real-world scenes, namely binocular disparity. Binocular disparity can be used by the visual system when processing the three-dimensional structure of the visual world. In particular, the presence of disparity can be of help when observers have to recognize the three-dimensional structure of objects [18]. The same advantage is observed when rotated views of objects [19], [20], [21] and faces [22] have to be recognized.

There is evidence that observers can memorize the spatial layout of relatively simple scenes when this is directly relevant to the task, in particular they are able to detect changes in the scene spatial arrangement despite a rotation in the view. A viewpoint-change related performance cost is usually observed [23], [24], [25], [26], which might depend on whether the rotation is caused by the locomotion of the observer [27], [28].

Contrary to chromaticity and motion, no study to our knowledge has investigated whether the presence or absence of disparity affects the visual long-term memory for scene pictures. The presence of disparity could influence our long-term memory for pictures of scenes in at least two ways. At the encoding level it could favor the segmentation of objects in the scene, furthermore, it could contribute to the establishment of a detailed 3D representation of the scene, including the relative distances of objects from the observer [29], which could be stored in memory along with their color and form.

The contribution of binocular stereo to scene long-term memory could thus be generic, if binocular disparity would contribute for instance to better define the shape of the objects in the scene, or specific, if observers would be able to directly remember the binocular disparity associated with elements in the scene. Evidently, it is easier to extrapolate the three-dimensional structure of a scene from a 2D picture than it is to extrapolate its chromaticity from a grayscale picture, and there is no way one can infer observer motion from a still picture. Extremely rich monocular cues to the 3D structure of scenes, such as occlusions, illumination patterns and texture gradients are also present in 2D scene pictures. It could thus be the case that those monocular cues are sufficient to generate the quality of 3D scene structure stored in long-term memory. If so, binocular stereo would then not provide any specific information for the purpose of long-term memory storage, or possibly only a generic increase in the information encoded and retained in long-term memory.

In the first experiment we set out to assess the contribution of stereoscopic 3D information to the long-term memory for scenes, using a paradigm inspired by the study by Wichmann and colleagues [10], which includes separate learning and recognition sessions and the presentation of scenes from a limited number of categories. After finding no evidence for an enhanced recognition of stereo pictures of scenes containing cars, buildings and pictures of forest scenes, we tested our observers in a modified paradigm, using once again forest images. Only in this case we found a small increase in the recognition rate with stereo presentation. In the third and final experiment we tested directly whether observers retain a long-term trace of 3D presentation of the scenes. The results indicate that the presence of binocular disparity is not retained in long-term memory together with the identity of the scene.

## Experiment 1: Long-term Memory for Car, Building and Forest Images

In Experiment 1 we asked whether presenting scenes in 3D improves long-term memory performance. We used scenes belonging to three categories, i.e. scenes containing buildings, scenes containing cars and forest scenes. The rationale behind the choice of the stimulus categories was to vary the relative relevance of the spatial layout in the scenes and to vary the strength of binocular disparity signals. The first two categories contain mainly man-made objects in a urban setting and could be mainly identified based on the functional characteristics of the objects, whereas the forest scenes are completely deprived of man-made objects and we assumed that their memorization might be more strongly supported by a representation of the spatial layout. The building pictures contain objects which are comparatively more distant (usually beyond 5 meters) from the observer and their processing should be less affected by the weaker binocular disparity signal.

Furthermore, we manipulate presentation time. This allowed us on one side to prove the sensitivity of our paradigm using a manipulation which has been shown to affect long-term memory for pictures before [10]. We also speculated that a possible contribution of stereo could be limited to the case where enough processing time was available.

### Methods

Two groups of 28 students of the Justus-Liebig University of Giessen volunteered for participating in the study. The first group (23 females, mean age 22.9) was tested in the short-exposure condition, the second group (23 females, mean age 24.5) was tested in the long-exposure condition. Subjects in this experiment and in the following ones provided written informed consent in agreement with the Declaration of Helsinki. Methods and procedures were approved by the local ethics committee LEK FB06 at Giessen University (proposal number 2009-0008).

#### Stimuli

The stimuli were 192 3D pictures belonging to three categories: Houses, Cars and Forest scenes (64 pictures for each category).

The Pictures were taken with a Fujifilm Finepix W1 3D digital camera (Fujifilm Holdings Corporation, Tokyo, Japan). Images were first rescaled from 3648×2736 to 1000×750 pixels. Subsequently, the luminance pixel-wise mean and standard deviation for each RGB channel and picture were normalized to the mean and 25th percentile of the distribution in the original set, respectively. The stimuli were presented on a black background.

The pictures were shown on a 22-inch SyncMaster 2233 LCD Monitor (Samsung Group, Seoul, South Korea) running at 120 Hz. The monitor was viewed through Nvidia 3DVision shutter glasses (Nvidia Corporation, Santa Clara, CA), providing an effective frame-rate of 60 Hz.

The presentation of the stimuli was controlled using Matlab (MathWorks, Inc., Natick, MA) and the PsychToolbox [30].

Viewing distance was 100 cm and the pictures subtended 16.2×12.1° of visual angle.

At the end of the experiment all participants reported being clearly able to see the 3D structure of the pictures under our 3D stimulation conditions.

#### Procedure

The experiment consisted of a learning session directly followed by a recognition session.

**Figure 1:**
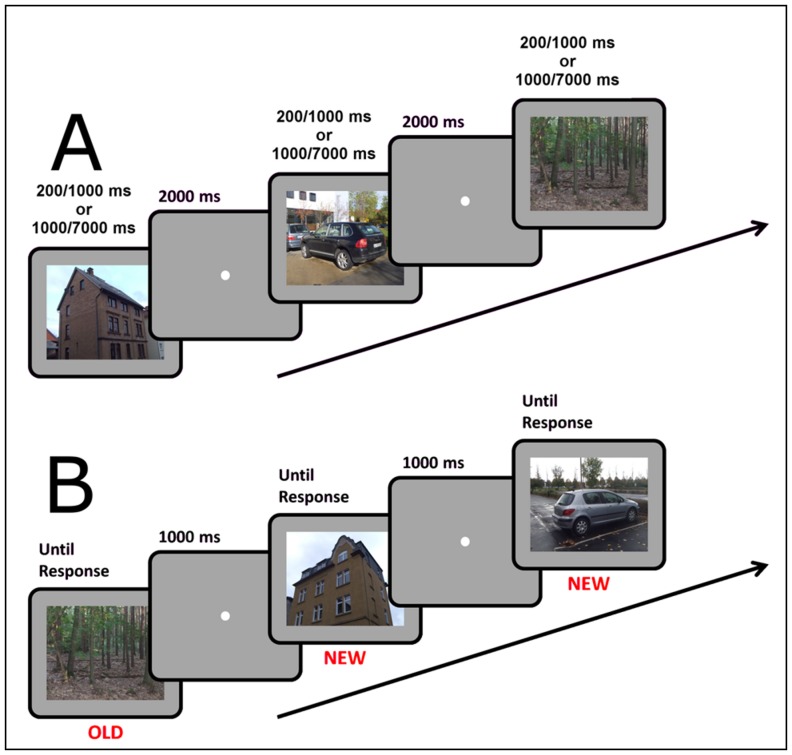
Experimental procedure in the Learning session (A) and in the Recognition session (B) of Experiment 1. In the Recognition session observers indicated whether they had seen the picture in the learning session (“old”) or whether they thought it had not been presented before (“new”).

In the learning session observers viewed half of the picture set (32 pictures for each category) in sequence. Each picture was shown for either 200 or 1000 ms to the observers of the short-exposure group and for either 1 or 7 seconds to the observers in the long-exposure group. Images were shown either in 2D or in 3D, followed by a 2 s interval during which only a fixation point was presented (Figure 1A). The category, exposure time and display type varied randomly from one picture to the next. Observers were instructed to look carefully at the pictures and to try to remember them in order to be able to recognize them in the subsequent session.

In the recognition session observers viewed the complete picture set in random sequence. Each picture was presented until the participant pressed one of two keys on a computer keypad (Figure 1B). The observers were instructed to press the right key to indicate that the image had been presented during the learning session (“old”) and the left key to indicated that the image had not been presented before (“new”). The keypress triggered the appearance of a fixation point for one second, which was followed by the presentation of the next picture. The observers were also informed that an equal number of “new” and “old” pictures would be presented in the recognition session.

Like in the learning session, half of the pictures were presented in 3D and half were presented in 2D. The display type for the old pictures was also the same in the two sessions, i.e. if a picture was presented in 3D in the learning session it was also presented in 3D in the recognition session and vice-versa. Like in the learning session, the display was randomly interleaved in the sequence.

### Results and Discussion

The responses collected in the recognition session were analyzed considering the “old” picture as a signal, and thus the correct recognition of an “old” picture as a Hit and the incorrect identification of a “new” picture as a False Alarm.

In the Short-Exposure group the overall Hit Rate was 53.47% whereas the overall FA rate was 31.96%. In the Long-Exposure group the overall Hit Rate was 60.82%, whereas the overall FA rate was 22.57%. This indicates that in both cases performance was better than chance level and far below perfection, avoiding floor and ceiling effects.

**Figure 2:**
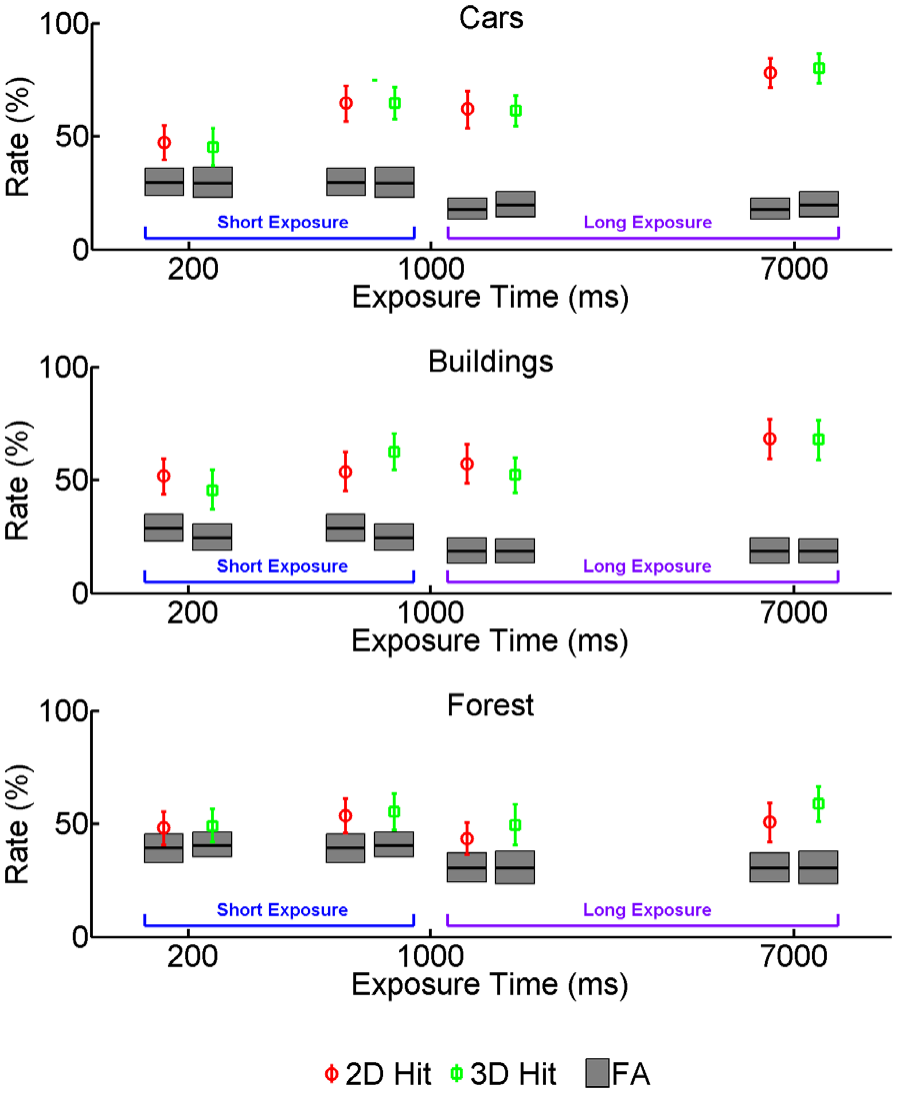
Average Hit Rate as a function of Display Type, Picture Category and Exposure Time in Experiment 1. Correct recognitions of old pictures are classified as Hits. The Blue and purple brackets indicate data from the Short Exposure and Long Exposure groups, respectively. Gray bars represent the corresponding average False Alarm Rate. Incorrect recognitions of new pictures are classified as False Alarms. Error bars are between-observer 95% confidence intervals of the mean.

We first analyzed the Hit and False Alarm rates separately, average values are depicted in Figure 2.

We also first analyze separately the data from the Short-Exposure Group and from the Long-Exposure Groups.

As for the Short-Exposure Group, a repeated-measure ANOVA on Hit Rate with Display Type (2D vs. 3D), Category (Cars vs. Buildings vs. Forest) and Exposure Time (200 vs. 1000 ms) as factors yielded a significant main effect of Exposure time (F(1,27) = 26.025, p<.001, ηp2 = .490) and a significant Exposure Time×Category interaction (F(2,54) = 4.256, p<.019, ηp2 = .136). The main effect of Category (F(1,27) = 0.758, p = .473, ηp2 = .027) was not significant and, crucially, none of the effects and interactions involving Display Type was significant (main effect: F(1,27) = .089, p = .768, ηp2 = .003, Display Type×Exposure Time interaction: F(1,27) = 2.522, p = .123, ηp2 = .085, Display Type×Category interaction: F(2,54) = .165, p = .848, ηp2 = .006, three-way interaction: F(2,54) = 2.099, p = .132, ηp2 = .085 ).

The False Alarm rate was analyzed with a repeated-measure ANOVA with Display Type (2D vs. 3D) and Category (Cars vs. Buildings vs. Forest) as factors (the factor Exposure Time is not defined for the False Alarm rate which is calculated from “new” pictures). This revealed a significant main effect of Category (F(2,54) = 12.659, p<.001, ηp2 = .32), whilst both the main effect of Display Type (F(1,27) = 0.395, p = .535, ηp2 = .01) and the two-way interaction (F(2,54) = .682, p = .51, ηp2 = .02) were not significant.

As for the Long-Exposure Group, a repeated-measure ANOVA on Hit Rate with Display Type (2D vs. 3D), Category (Cars vs. Buildings vs. Forest) and Exposure Time (1000 vs. 7000 ms) as factors yielded a significant main effect of Category (F(2,54) = 24.701, p<.001, ηp2 = .477) and a significant main effect of Exposure Time (F(1,27) = 65.471, p<.001, ηp2 = .708). Contrary to what we observed in the Short Exposure Group, the Exposure Time×Category interaction was not significant (F(2,54) = 1.994, p = .146, ηp2 = .068). Like in the Short Exposure Group, none of the effects and interactions involving Display Type was significant (main effect: F(1,27) = . 553, p = .463, ηp2 = .020, Display Type×Exposure Time interaction: F(1,27) = 2.498, p = .091, ηp2 = .084, Display Type×Category interaction: F(2,54) = .165, p = .848, ηp2 = .006, three-way interaction: F(2,54) = .036, p = .965, ηp2 = .001 ).

Like in the case of the Short-Exposure Group, the False Alarm rate in the Long-Exposure Group was analyzed with a repeated-measure ANOVA with Display Type (2D vs. 3D) and Category (Cars vs. Buildings vs. Forest) as factors. This revealed a significant main effect of Category (F(2,54) = 14.741, p<.001, ηp2 = .35), whilst both the main effect of Display Type (F(1,27) = 0. 197, p = .660, ηp2 = .01) and the two-way interaction (F(2,54) = . 178, p = .837, ηp2 = .01) were not significant.

The 1000 ms exposure time was common to both the Short- and Long-Exposure groups. In order to exploit the full statistical power of our sample we analyzed the corresponding Hit Rate data in an overall ANOVA with Category (Cars vs. Buildings vs. Forest) and Display Type (2D vs. 3D) as within-subject factors and Group (Short-Exposure vs. Long Exposure) as a between-subject factor. The effect of Category was significant (F(2,108) = 10.759, p<.001, ηp2 = .166), whereas all other effects and interactions were not significant (main effect of Display Type: F(1,54) = .817, p = .370, ηp2 = .015, main effect of Group: F(1,54) = 1.740, p = .193, ηp2 = .031, Display Type×Group interaction: F(1,54) = .659, p = .420, ηp2 = .012, Group×Category interaction: F(2,108) = .519, p = .597, ηp2 = .010, Display Type×Category interaction: F(2,108) = .479, p = .620, ηp2 = .009, three-way interaction: F(2,108) = 2.195, p = .116, ηp2 = .039 ).

For comparison, we also performed an ANOVA with Category (Cars vs. Buildings vs. Forest) and Display Type (2D vs. 3D) as within-subject factors and Group (Short-Exposure vs. Long Exposure) as between-subject factor on the False Alarm rate. The main effect of Category (F(2,108) = 26.993, p<.001, ηp2 = .333) and the main effect of Group (F(1,54) = 8.618, p = .005, ηp2 = .138) were significant, whereas all other effects and interactions were not significant (main effect of Display Type: F(1,54) = .031, p = .862, ηp2 = .001, Display Type×Group interaction: F(1,54) = .585, p = .448, ηp2 = .011, Group×Category interaction: F(2,108) = .233, p = .793, ηp2 = .004, Display Type×Category interaction: F(2,108) = .588, p = .557, ηp2 = .011, three-way interaction: F(2,108) = .378, p = .686, ηp2 = .007).

**Figure 3:**
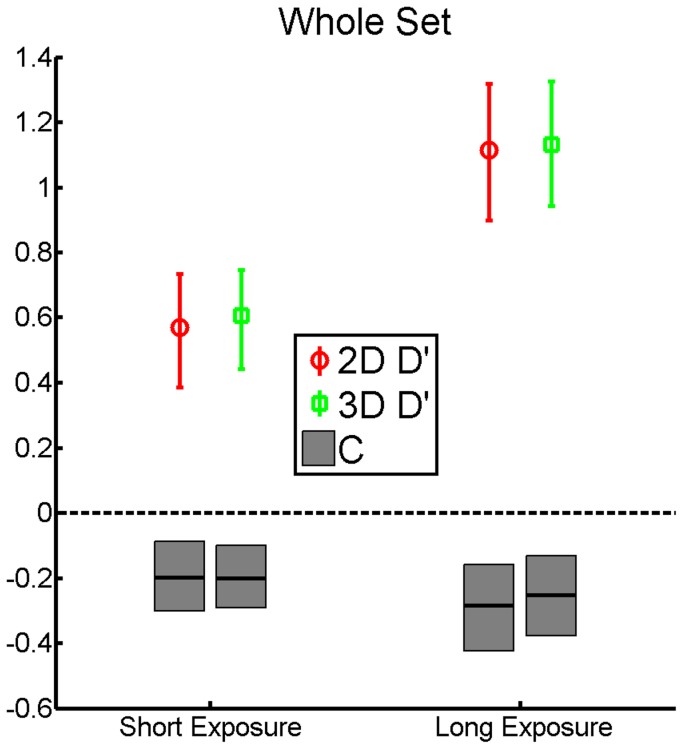
Average d′ (red and green bars) and c (gray bars) values as a function of Display Type in the two Groups. Error bars are between-observer 95% confidence intervals of the mean. Sensitivity is not influenced by stereo presentation but increases significantly with longer exposure. In both groups observers are biased not to report 3D presentation (c values are on average negative).

In a second analysis we computed sensitivity (d´) and criterion (c) measures from our results. Given that the Exposure Time is undefined for the “new” pictures, d´ and c cannot be analyzed as a function of this factor. Moreover, we decided to collapse the three categories in order to calculate the rates from a larger trial number thus reducing the necessity to deal with infinite d´ or c values. One observer in the Long-Exposure group did not produce any false alarms in the 2D Display. A False Alarm Rate equal to half a trial (1.04%) was assumed in this case. The average d´ and c values are depicted in Figure 3. Sensitivity was almost twice as high in the long-exposure group as compared to the short-exposure group. Nonetheless, in neither case d´scores indicate an advantage for stereo presentation. Moreover, in both groups the criterion values were negative, indicating that observers tended to identify the pictures as new.

The d′ values were submitted to an ANOVA with Display Type (2D vs. 3D) as a within-subject factor and Group (Short-Exposure vs. Long Exposure) as a between-subject factor. Not surprisingly, given the significant increase in hit rate as a function of exposure time in each individual group, the main effect of Group was significant: F(1,54) = 19.242, p<.001, ηp2 = .263), whereas the main effect of Display Type (F(1,54) = .268, p = .606, ηp2 = .005) and the Display Type×Group interaction (F(1,54) = .032, p = .858, ηp2 = .001) were not significant.

The same analysis performed on c values failed to provide any significant result (main effect of Group: F(1,54) = 1.396, p = .243, ηp2 = .025, main effect of Display Type: F(1,54) = 1.321, p = .255, ηp2 = .024, Display Type×Group interaction: F(1,54) = .079, p = .780, ηp2 = .001).

Overall, the results of the first experiment suggest that presenting images in 3D might not enhance the probability that they will be recognized from long-term memory. This was the case for the car and building scene images, whereas the level of performance in the forest images might have been too low thus masking the potential benefit due to stereo presentation.

The recognition of car and building images might have been mediated primarily by the recognition of diagnostic man-made objects within the scene, whose storage might in principle not be supported by a visual code. The forest images, on the contrary, are primarily identified by their spatial arrangement and it is crucial to understand whether binocular stereo can be of any help in this specific image category.

## Experiment 2: Forest Images

In the first experiment memory performance was much lower than one could expect based on the capacity of long-term visual memory for scenes [1], [2]. A possible reason might be related to the fact that memory for scenes is very often supported by a conceptual representation, which involves some form of distinctive categorization of the picture [7], [31]. Since we always used relatively similar items belonging to the same category as targets and distractors, this form of long-term memory for pictures was neutralized.

This was particularly true for the forest category, where observers were faced with extremely similar distractors. This however might indicate that a potential advantage with stereo presentation was masked by floor effects. In Experiment 2, in order to get a more stable index of memorization performance, we increased the number of pictures that were presented. In order to increase the recognition performance we used a 2AFC task rather than an old/new task. In order to gain more statistical power we also decided to simplify the experimental design by only presenting our images for 7 seconds, the condition in which the results from Experiment 1 seemed to suggest a possible stereo advantage. In order to keep the duration of the experiment, and thus the retention period, comparable to Experiment 1, we also presented our observers with a set of office scenes.

### Methods

One groups of 28 students of the Justus-Liebig University of Giessen (23 females, mean age 23.9) volunteered for participating in the study.

#### Stimuli

The stimuli were 80 3D pictures depicting forest scenes (taken from the larger database to which the images used in Experiment 1 belonged) and 80 images of office scenes.

Image processing and stimulus presentation were conducted as in Experiment 1 with the difference that the size of the pictures was reduced to 60% of the original size in the recognition session.

At the end of the experiment all participants reported being clearly able to see the 3D structure of the pictures under our 3D stimulation conditions.

#### Procedure

The experiment consisted of a learning session directly followed by a recognition session.

**Figure 4:**
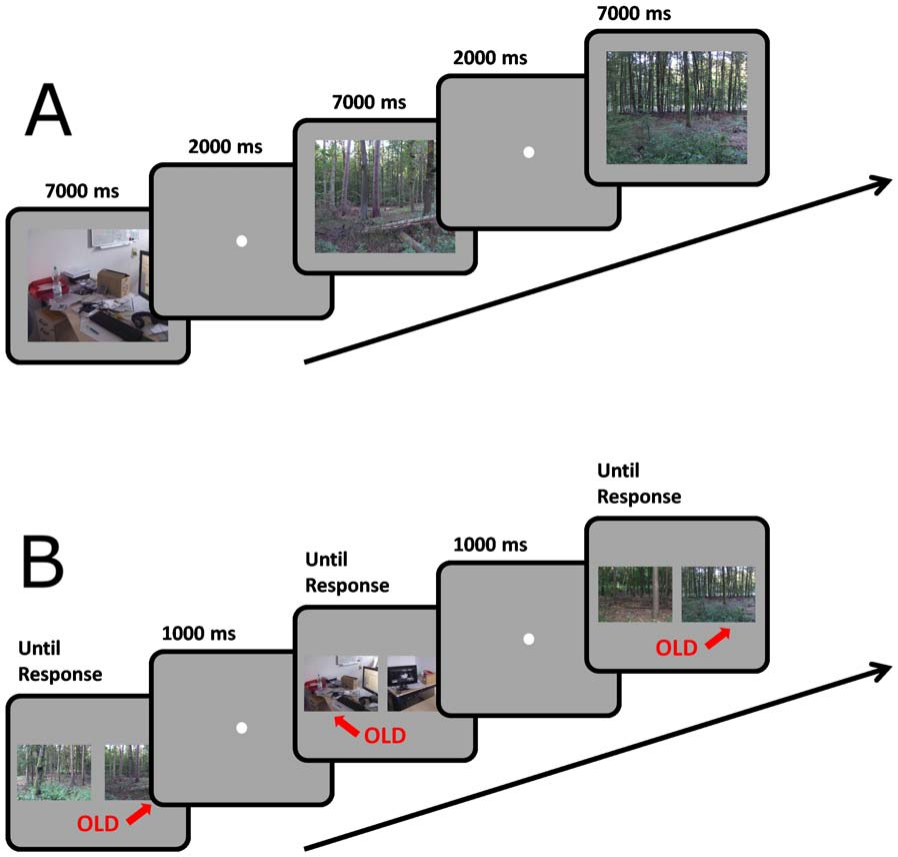
Experimental procedure in the Learning session (A) and in the Recognition session (B) of Experiment 2. In the Recognition session observer indicated which of the two images had been presented in the previous session (“old” picture). Target images were always paired with a distractor from the same category.

In the learning session observers viewed half of the picture set (80 pictures) in sequence. Each picture was shown for 7000 ms to the observers. Images were shown either in 2D or in 3D, followed by a 2 s interval during which only a fixation point was presented (Figure 4A). The display type varied randomly from one picture to the next. Observers were instructed to look carefully at the pictures and to try to remember them in order to be able to recognize them in the subsequent session.

In the recognition session observers viewed again all the pictures which were presented in the learning session in random order. Each old picture was presented together with a new picture from the same category. The old picture and the new one were presented randomly on the right and left side of the screen (Figure 4B). The pictures remained on the screen until the participant pressed one of two keys on a computer keypad. The observers were instructed to press the right or left key to indicate which of the images they thought was old. The keypress triggered the appearance of a fixation point for one second, which was followed by the presentation of the next couple of pictures. The observers were informed that each pair contained a new and an old picture.

The display type was the same for both pictures in a pair and corresponded to the display type of the old picture in the learning session.

### Results and Discussion

The observers’ performance with office scenes was substantially at ceiling, in both Display Types 10 observers out of 28 had 100% accuracy. The corresponding data were not evaluated further.

**Figure 5:**
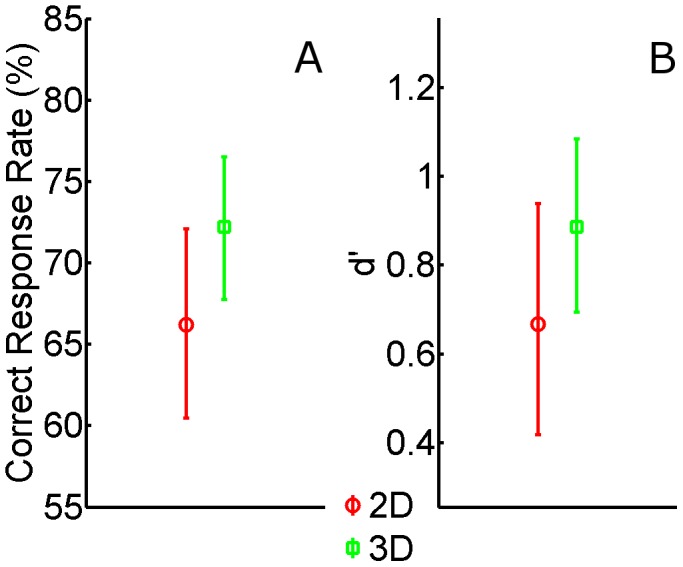
Average performance in terms of percentage correct responses and d` in Experiment 2, as a function of Display Type. Error bars are between-observer 95% confidence intervals of the mean. The 6% increase in the rate of correct responses with 3D presentation was statistically significant.

The responses from the observers with forest scenes are reported in Figure 5, both in terms of percent correct answers and d´. Evidently, in this paradigm observers perform largely over chance with forest images. Crucially, observers identify correctly 6% more stereo presented pictures as compared to non stereo presentation, i.e. on average 2.4 pictures out of 40.

A paired-t test on d´ scores revealed that the correct recognition was higher for 3D images as compared to 2D presentation (t(27) = 2.053; p<0.05).

The significant effect of Display Type corresponds to an average increase in the correct response rate of 6.0%, which, in the 2AFC recognition task, implies an increase of 12% in the number of actually recognized pictures. Overall, the results of Experiment 2 indicate that when enough time is available in order to encode the images, forest scenes, which are poorly identified by the objects they contain, can be remembered better with binocular stereo presentation.

Overall, the result is compatible with the tendency which we observed for an increased hit rate in the recognition of forest pictures in Experiment 1 when they were presented for 7 seconds in the learning phase. We are inclined to interpret the fact that the effect was significant in Experiment 2 as a consequence of the reduced task difficulty in the forced-choice task, i.e. a floor effect might have reduced the enhancement in Experiment 1. Task difficulty however cannot explain the lack of any enhancement in the memory for building and car scene stereo pictures in Experiment 1. Average d´in Experiment 2 was 0.77, whereas in Experiment 1, using the FA rate shared between short and long duration trials we estimated a d’ value of 1.06 for the 1 second presentation averaging over groups, display types and the two categories. Thus, we can be confident that the lack of any contribution of stereo presentation to the long-term memory of building and car scenes in Experment 1 was not simply due to the fact that the task was too difficult, as we think was the case for the forest images.

## Experiment 3: Testing the Memory for Stereo Presentation

The results from the first two experiments indicate that stereo presentation enhances long-term visual memory for scene images only when observers are allowed to encode the scene for an extremely long time and when the scenes are maximally identified by their spatial arrangement. The lack of any advantage for 3D presentation in Experiment 1 could have two interpretations. On one hand, the observers might not have retained a trace of the stereo quality of the pictures, alternatively, the observers might have retained a memory of whether the display was 3D but this could be not relevant for picture recognition, which could be mediated by non-spatial attributes of the picture. In the third and last experiment we explicitly test our observerś memory for the pictureśdisplay type in a modified version of Experiment 1.

### Methods

One groups of 28 students of the Justus-Liebig University of Giessen (18 females, mean age 24.5) volunteered for participating in the study.

#### Stimuli

The stimuli were 96 3D pictures randomly sampled from the ones used in Experiment 1 (32 pictures for each category).

Image processing and stimulus presentation were conducted as in Experiment 1 with the difference that the size of the pictures was reduced to 60% of the original size in the recognition session.

At the end of the experiment all participants reported being clearly able to see the 3D structure of the pictures under our 3D stimulation conditions.

#### Procedure

The experiment consisted of a learning session directly followed by a recognition session.

**Figure 6:**
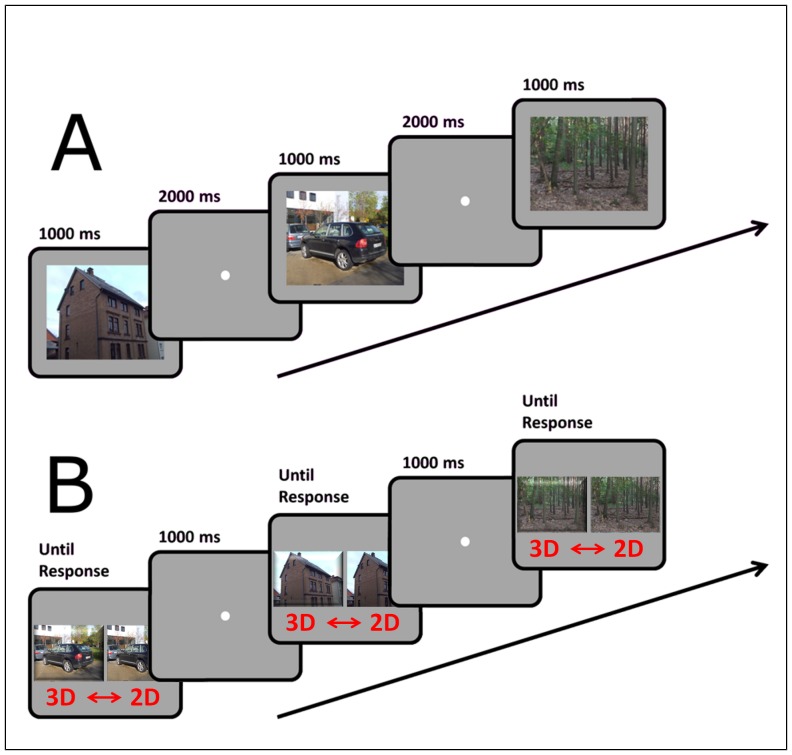
Experimental procedure in the Learning session (A) and in the Recognition session (B) of Experiment 3. The learning session was identical to the one of Experiment 1 (with 1 s exposure). In the Recognition session observer indicated the type of the display in which the image had been presented in the previous session by choosing the corresponding target. For each observer the 3D version of the image was always presented on the same side of the screen.

The learning session followed exactly the same procedure as in Experiment 1 (Figure 6A). Observers were instructed to look carefully at the pictures and to try to remember them in order to be able to recognize them in the subsequent session. At the end of the learning session the observer was instructed to call the experimenter, who explained the actual task for the memory testing session. Observers were informed that their task would not be the one of recognizing the pictures but to indicate for each picture whether they thought it had been presented in 2D or 3D.

In the memory test session a 3D version and a 2D version of each picture were presented on the right and left side of the screen (Figure 6B). The association between the display type and the side of the screen was alternated between observers. The pictures remained on the screen until the participant pressed one of two keys on a computer keypad. The observers were instructed to press the right or left key to indicate in which display type they thought the image had been presented in the learning session. The keypress triggered the appearance of a fixation point for one second, which was followed by the presentation of the next couple of pictures. The observers were informed that each pair contained a new and an old picture.

### Results and Discussion

**Figure 7:**
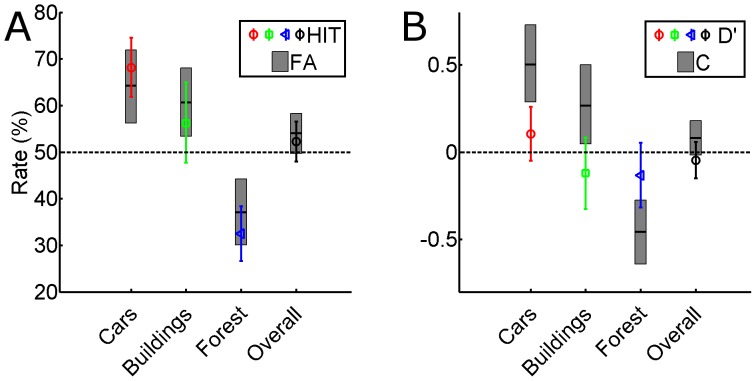
Results from Experiment 3 in terms of Hit and False Alarm rates (A) and of Sensitivity and Criterion (B). Hits are defined as the correct indication that an image had been presented in 3D. Data are presented separately for each category of pictures (colored bars) and for the overall data (black bars). Error bars represent between-observer 95% confidence intervals of the mean. Sensitivity (d`) did not differ from 0 in any of the single categories nor in the overall data. Observers were biased to report that car and building images had been presented in 3D, whereas they tended to report that forest images had been presented in 2D.

The responses of the observers are depicted in Figure 7A. Although the task is framed as a 2AFC, it is in essence a present-absent task. Each trial can be identified as signal present/absent depending on the picturés display type in the learning sessions and the observerś choice is coded along this dimension (i.e. they had to report whether they thought the picture had been presented in 2D or 3D. This allowed us to define Hits, False Alarms, Sensitivity and Criterion. The Hit and False Alarm rates (considering 3D presentation as the signal) are similar within each picture category and in the overall results, although the tendency to report (correctly or falsely) that a picture had been presented in 3D differs between categories. In particular, observers were likely to report that forest scenes had been presented in 2D whereas they tended to report that car and house pictures had been presented in 3D.

The results have been converted to sensitivity (d´) and criterion (c) for further analysis (Figure 7B). Limitedly to the Car category one observer had a False Alarm Rate of 100% and another observer had both a False Alarm and a Hit Rate of 100%. In all three cases an error rate equal to half trial (3.1%) was assumed for d` and c calculation.

One-sample t-tests were performed on d` and c values in order to test whether observers were able to recollect the original display type and whether they had a bias to report one of the two types. Sensitivity (d`) was neither significantly different from 0 overall (t(27) = .848, p = .404) nor in the single categories (Cars: t(27) = 1.299, p = .205; Buildings: t(27) = 1.140, p = .264; Forest: t(27) = 1.393, p = .174). The overall criterion was not different from 0 (t(27) = .848, p = .404).

The observers’ sensitivity regarding stereo presentation is limited by their ability to retain a trace of the picture in the first place, i.e. it is unlikely that observers can recollect the display type of a given picture without recognizing the picture in the first place. Unfortunately, only a rough estimate of the observerś sensitivity in recognizing the pictures can be obtained from the results of Experiment 1. In general, from Figure 3 we can deduce that the average d` value as observed in the short-exposure group (0.59) was around three times larger than the average of the lower arm of the corresponding confidence interval (0.17). Obviously, the sensitivity in the short-duration condition of Experiment 1 is a conservative estimate of the expected sensitivity in Experiment 3, given that half of the pictures were encoded with a shorter presentation time and that observers were not exposed to new distractor pictures during the recognition test phase. Overall, this suggests that, despite the level of noise, even if the observers had recollected correctly the display type of only a subset of the pictures that they could recognize, they would still have provided non-zero sensitivity in Experiment 3.

Observers also had a quite strong tendency to report that house and car images had been presented in 3D, whereas they tended to report that the forest images had been presented in 2D. In Greene and Olivás [32] terminology, the three categories of scenes differ markedly in their structural properties. In particular, car and house images have a much larger expansion as compared to forest images, also due to the fact that in order to maximize the disparity gradients most buildings and cars were captured from rather angled points of view. The expansion in the scene might have conveyed a rather strong impression of three-dimensionality even from a 2D presentation.

## Discussion

In a delayed recognition task, we investigated whether the presence of binocular disparity can improve the long-term memory for scenes. The results indicate that this is only the case when an extremely long time is available to encode pictures and when the spatial layout of the scene is prominently relevant to distinguish target pictures from foils belonging to the same category. Specifically, we only found a stereo enhancement of the long-term memory for forest pictures which had been studied for 7 seconds. When observers memorized images of houses or vehicles, which are more likely to be encoded conceptually, the recognition performance, both in terms of Hit Rate and in terms of sensitivity was no better for stereo pictures as compared to 2D scene pictures.

Based on the fact that we were able to demonstrate a highly significant effect of Exposure Time, thus replicating a finding which was reported by Wichmann and colleagues [10], we believe that the paradigm we used in Experiment 1 was powerful enough to detect relevant modulations of recognition performance. Still, the fact that stereo presentation did not enhance the recognition rate of car and house images does not per se imply that stereo was ignored while encoding the pictures. Indeed, we have indications that stereo presentation had a differential effect depending on the scene category (i.e. it was only advantageous for forest pictures), thus the lack of a stereo recognition advantage might be related to the fact that pictures containing distinctive artifacts are recognized based on a conceptual rather than visual code. Recognizing scenes through distinctive objects might be the default way, especially when enough time is available to proceed from the encoding of the general structure of the scene to a more detailed description [33].

In Experiment 3 we asked explicitly whether our observers retained a trace of whether the pictures had been presented in stereo. This did not seem to be the case, even for the scene categories which proved to be well recognizable in Experiment 1.

Overall, the results indicate that binocular stereo is only useful while encoding scene pictures to retain in long-term memory if the tree-dimensional structure of the scene is crucial for the task and when enough time is available. Otherwise, the trace which is retained from a 3D picture is equivalent to the trace retained from a 2D picture, both in the information it conveys for the purpose of recognition and in its visual quality.

This result parallels the general finding that the contribution of binocular disparity to visual perception and memory for comparatively simple objects and faces is maximally evident when their 3D structure is made explicitly relevant by the task, e.g. when rotated views have to be recognized [18], [19], [20], [21], [22].

Conversely, the relatively limited enhancement of long-term memory for stereo pictures of scenes is in contrast with the robust advantages induced by chromaticity [10], [11], [12] and motion [14]. One possible explanation might relate to the fact that the information conveyed by chromaticity may not be easily recovered from a grayscale picture, given that chromaticity is relatively independent from luminance in natural images [34], and for instance observer motion is not coded at all in static images. The depth arrangement of objects in a scene can instead be recovered from a number of monocular cues, such as occlusions, illumination gradients, perspective. This might undermine the relevance of stereo when a coarse coding of the spatial structure of the scene is sufficient.

The question of what specific mechanism supports the stereo-viewing enhancement of long-term memory performance for forest pictures remains open. On one side, the fact that this enhancement is particularly evident when images are presented for 7 seconds suggests that visual segmentation might probably not be the most important factor and that observers stored a better representation of the relative depth of one or more elements within the scene, which later helped to distinguish the target scenes from foils containing conceptually similar elements but located on different depth planes. In this sense, binocular disparity would contribute specific information to memory. On the other side, we cannot exclude that the presence of stereo might have favored the initial encoding of the scene simply by enhancing the segmentation of the different elements which in turn are learned. Binocular disparity would then not constitute a specific source of information for the memory task, as is the case with chromaticity and motion. This would be consistent with the results of Experiment 3, showing that observers do not retain a trace of the compelling three-dimensionality impression which was induced by binocular disparity while they were viewing the scene picture.

If it is true that binocular disparity contributes a specific source of information to long-term memory, and this contribution becomes relevant when most cues to recognition based on form, chromaticity and semantics are degraded to a large extent, we can predict that a benefit due to binocular stereo could emerge also for urban scenes, as long as they contain a limited set of objects distinguished by their spatial arrangement, rather than by their color, shape and functional meaning.

In general, future research should aim at testing the contribution of binocular disparity to visual long-term memory in a larger sample of scene categories, in order to establish whether the enhancement we found in the case of forest images is a rare exception or a widespread phenomenon.
